# Impact of Gamma Irradiation and Kale Leaf Powder on Amino Acid and Fatty Acid Profiles of Chicken Meat under Different Storage Intervals

**DOI:** 10.3390/molecules27238201

**Published:** 2022-11-24

**Authors:** Waseem Khalid, Muhammad Sajid Arshad, Gulzar Ahmad Nayik, Saleh Alfarraj, Mohammad Javed Ansari, Raquel P. F. Guiné

**Affiliations:** 1 Department of Food Science, Faculty of Life Sciences, Government College University, Faisalabad 38000, Pakistan; 2 Department of Food Science & Technology, Government Degree College Shopian, Srinagar 192303, India; 3 Zoology Department, College of Science, King Saud University, Riyadh 11451, Saudi Arabia; 4 Department of Botany, Hindu College Moradabad, Mahatma Jyotiba Phule Rohilkhand University Bareilly, Moradabad 244001, India; 5 CERNAS Research Centre, Polytechnic Institute of Viseu, 3504-510 Viseu, Portugal

**Keywords:** chicken meat, gamma rays, kale leaf powder, amino acids, fatty acids

## Abstract

The present study was planned to determine the effect of kale leaf powder and gamma rays on variations in the pH, amino acid and fatty acid profiles of chicken meat at different storage intervals. Significant changes (*p* ≤ 0.05) in the pH, amino acid and fatty acid profiles of chicken meat following different treatments (KLP (1% and 2%) and gamma irradiation (3k Gy)) were reported at 0, 7 and 14 days of storage. The pH value of the chicken meat sample decreased with the addition of kale leaf powder, whereas the value increased following a gamma irradiation dose of 3 kGy and with the passage of time. During different storage intervals, the minimum reduction in the amino acid and fatty acid quantities in the chicken meat samples was reported after gamma irradiation treatment. However, with the addition of KLP, the amount of amino acids and fatty acids in the chicken meat samples increased. Conclusively, the pH was observed to be reduced in the meat following combined treatment (irradiation + KLP), whereas the 2% KLP treatment improved the amino acid and fatty acid profiles of the chicken samples.

## 1. Introduction

Meat is a rich source of protein that contributes to the development of various human body parts [1]. Meat is also composed of water, fat, vitamins (B1, B2, B6 and E), minerals (selenium, iron and zinc) and bioactive compounds. It provides different nutrients and phytochemicals to humans (alpha-lipoic acid, conjugated linoleic acid, glutathione, taurine, creatine, l-carnitine and choline) [2]. White meat, such as chicken, is the most consumed type of meat in the world. Worldwide, chicken is considered as a common type of poultry because it is low cost and has a high nutrient profile [3].

The safety and quality of meat products must be high to ensure a healthy diet. The demand for food preservation has been increasing with time. Previously, thermal processing techniques were used globally for food preservation. These preservation techniques resulted in many changes in the products, such as the creation of an aroma, the softening of texture, protein coagulation and swelling of starch [4]. However, nowadays, most consumers worry about health carcinogenic problems caused by the thermal processing of meat and meat products [5]. However, some non-thermal processing technologies that use gamma irradiation are being used to produce safe and high-quality meat [6]. Gamma irradiation (cesium 137 or cobalt 60) enhances the meat product’s shelf life by inhibiting the growth of bacteria [7]. These rays eliminate the pathogenic bacteria from meat products and aid in improving human health [8]. Gamma irradiation is approved by the Food and Drug Administration and is used in meat processing to increase consumer satisfaction, improve nutrition, and increase shelf life and product quality. Due to their high penetration power and energy, gamma rays work on a cellular level in food particles [9]. Chicken meat can be exposed to doses up to 7 kGy increase the shelf life of meat products and to ensure they are suitable for human consumption [10]. During the ionization process, radiolysis in water generates a number of free radicals. Furthermore, the oxidation process starts in meat products due to these free radicals. Some natural plants are composed of biologically active compounds that aid in inhibiting the oxidation reactions during processing [11].

Nowadays, plant-based foods are required in our daily diets, in addition to other foods. Among other leafy vegetables, kale is a leafy vegetable that belongs to the cabbage family. It provides different nutrients (fatty acids, amino acids, vitamins and minerals) and phytochemicals (polyphenols, glucosinolates and carotenoids) to humans [12]. Kale is composed of biologically active components, such as phytochemicals, antioxidants, fiber, and large amounts of vitamins and trace elements [13]. The antioxidants in kale include tocopherols, beta-carotene and ascorbic acid, while the phytochemicals in kale include polyphenols, lutein and zeaxanthin [14]. The synergy between gamma irradiation and plant-based bioactive compounds contributes to the safety and quality of meat and meat products [15,16].

The current study was designed to measure the effects of different gamma irradiation doses (3 kGy) and kale leaf powder (1% and 2%) on the pH, amino acid and fatty acid profiles of chicken meat. The chicken samples were treated with kale leaf powder with and without gamma rays to check the variations in fatty acid and amino acids profile at different storage intervals.

## 2. Results

### 2.1. pH

pH is a basic parameter that is closely related to the quality of food and food products. It is also a vital factor that affects the stability of the bioactive compounds in meat and meat products [17].

**Figure 1:**
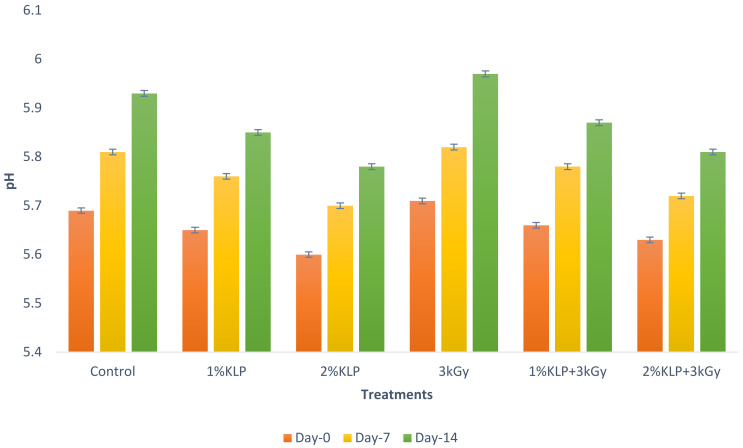
pH value of chicken meat treated with kale leaf powder and gamma irradiation at different storage intervals.

Significant changes (*p* ≤ 0.05) were observed in chicken meat with respect to different storage intervals and treatments. For the effect of storage on chicken meat, a high PH value of 5.97 ± 0.03 at day 14 was reported, while a lower value (5.60 ± 0.03) was reported at the start of storage. The kale leaf powder affected the pH results in chicken meat. For 2% Kale Leaf Powder (KLP), the lowest pH value was 5.60 ± 0.03, whereas the irradiation dose increased the pH of chicken meat (Figure 1).

A high PH value of 5.93 ± 0.04 was reported at day 14 and a lower value of 5.69 ± 0.04 was reported at day 0 in the control sample of chicken meat. At 3 kGy, the pH value of chicken meat was observed to increase as compared to the control sample, while a minor reduction was observed with the addition of kale leaf powder to the meat. Moreover, for 1% KLP, a high value of 5.85 ± 0.03 was reported and a lower value of 5.60 ± 0.03 was reported for 2% KLP. However, for 1% KLP + 3 kGy and 2% KLP + 3 kGy, the average pH values were 5.66 ± 0.03 and 5.63 ± 0.04, respectively, at day 0.

### 2.2. Hydrolyzed Amino Acids (HAAs) in Irradiated and Kale Leaf-Treated Chicken Meat

It was observed that the quantities of HAAs in chicken meat were significantly different (*p* ≤ 0.05) during different treatments. The quantities of essential and nonessential amino acids in the chicken patties ranged from 6487 ± 235 to 7175 ± 305 and 7216 ± 172 to 7721 ± 218 mg/100 g, respectively, for different treatments. In the chicken patties, the maximum quantity of essential amino acids was 7175 ± 305 mg/100 g for 2% KLP, while the lowest quantity was 6487 ± 235 for 3 kGy.

The average amount of arginine, histidine, isoleucine, leucine, lysine, methionine, phenylalanine, threonine and valine ranged from 985 ± 42, 410 ± 15, 499 ± 23, 1185 ± 20, 1270 ± 19, 372 ± 21, 623 ± 30, 623 ± 40 and 517 ± 25 to 1089 ± 43, 466 ± 34, 578 ± 40, 1259 ± 51, 1380 ± 35, 414 ± 24, 687 ± 18, 712 ± 28 and 590 ± 32 mg/100 g, respectively, for the irradiation and non-irradiation treatments. Lysine was recorded as one of the most prevalent amino acids, whereas histidine was recorded as the least prevalent. The average amount of aspartic acid, alanine, cysteine, glutamic acid, glycine, proline, serine and tyrosine ranged from 1499 ± 30, 982 ± 18, 42 ± 14, 2343 ± 26, 709 ± 18, 556 ± 14, 574 ± 20 and 538 ± 32 to 1583 ± 20, 1019 ± 40, 67 ± 10, 2413 ± 30, 778 ± 25, 632 ± 35, 649 ± 30 and 610 ± 28 mg/100 g, respectively. A high amount of glutamic acid was observed in the chicken sample, while a low quantity of cysteine was observed.

Kale leaf powder and the irradiation dose affected the chicken meat samples, as shown in Table 1. At 3 kGy, the amount of essential amino acids decreased, whereas, by enhancing the concentration of KLP, the amount of essential amino acids was observed to be high in the chicken samples. A higher amount of essential amino acids was observed for 2% KLP as compared to the control samples, while lower amounts of amino acids were recorded for the treatments 1% KLP + 3 kGy and 2% KLP+ 3 kGy. The irradiation and non-irradiation treatments affected the chicken meat samples, as shown in Table 1. At 3 kGy, the amount of non-essential amino acids decreased whereas, by enhancing the concentration of KLP, the amount of non-essential amino acids was observed to be high in the chicken samples. A higher quantity of non-essential amino acids was observed for 2% KLP as compared to the control samples, while the quantities of amino acids were low following the treatments 1% KLP + 3 kGy and 2% KLP+ 3 kGy, as compared to the control samples.

### 2.3. Free Amino Acids in Irradiated and Kale Leaf-Treated Chicken Meat

During different storage intervals and treatments, significant changes (*p* ≤ 0.05) were observed in the quantities of free amino acids in the chicken meat. The mean quantities of free amino acids in the chicken meat samples are given in Table 2. The amount of essential amino acids did not change significantly in chicken meat following different treatments, while changes were observed in the amount of non-essential amino acids.

The mean quantities of essential amino acids (free amino acids) ranged from 112.61 ± 0.37 to 134.89 ± 0.55 mg/100 g. At day 0, the maximum amount of essential amino acids was recorded, while the lowest quantity was found at day 14 following different treatments. In the chicken meat samples, during different storage intervals and treatments, the amount of arginine, histidine, isoleucine, leucine, lysine, methionine, phenylalanine, threonine and valine ranged from 65.69 ± 0.08 to 86.81 ± 0.15, 1.42 ± 0.02 to 1.73 ± 0.03, 4.10 ± 0.03 to 5.59 ± 0.05, 9.75 ± 0.05 to 11.68 ± 0.08, 0.45 ± 0.01 to 2.75 ± 0.03, 3.14 ± 0.02 to 3.82 ± 0.04, 5.17 ± 0.04 to 6.08 ± 0.05, 8.19 ± 0.02 to 10.00 ± 0.05 and 8.99 ± 0.05 to 10.99 ± 0.04 mg/100 g, respectively. Among all the essential amino acids, arginine was found in high quantities in the chicken meat, whereas histidine was found in low quantities.

The average mean quantities of non-essential amino acids (free amino acids) ranged from 152.01 ± 0.23 to 168.89 ± 0.37 mg/100 g. At day 0, the highest amount of non-essential amino acids was observed, while the lowest quantity was recorded at day 14. In the chicken meat samples, during different storage (0, 7 and 14 d) intervals and treatments, the following range of alanine, cysteine, aspartic acid, glutamic acid, glycine, proline, serine and tyrosine quantities was observed: (34.99 ± 0.06–40.01 ± 1.02), (0.34 ± 0.03–0.65 ± 0.03), (0.16 ± 0.01–0.68 ± 0.02), (67.16 ± 0.05–70.84 ± 0.09), (13.01 ± 0.03–14.87 ± 0.05), (18.73 ± 0.03–21.22 ± 0.05), (10.53 ± 0.05–12.82 ± 0.01) and (3.72 ± 0.03–6.68 ± 0.03), respectively. Among all the non-essential amino acids, the amount of glutamic acid was found to be high in chicken meat, whereas cysteine was found in low quantities.

The average mean quantities of derivative amino acids (free amino acids) ranged from 91.9 ± 0.31 to 107.68 ± 41 mg/100 g. At day 7, the highest amount of derivative amino acids was observed, while the lowest quantity was recorded at day 14. In the chicken meat samples, during different storage (0, 7 and 14 d) intervals and treatments, the following range of anserine, carnosine, cystathionine, ethanol amine, hydroxy proline, sarcosine, α-amino adipic acid, α-amino-n-butyric acid, β-alanine, γ-amino-n-butyric acid, 1-methylhistidine and 3-methylhistidine quantities was found: 1.14 ± 0.01–1.32 ± 0.02, 78.22 ± 0.07–87.66 ± 0.05, 1.15 ± 0.01–1.51 ± 0.03, 0.39 ± 0.01–1.88 ± 0.04, 2.98 ± 0.05–3.34 ± 0.02, 0.23 ± 0.01–0.43 ± 0.01, 1.35 ± 0.05–1.81 ± 0.05, 0.19 ± 0.01–0.31 ± 0.02, 0.23 ± 0.02–2.39 0.03, 0.48 ± 0.01–1.33 ± 0.01, 1.04 ± 0.03–2.25 ± 0.04 and 0.34 ± 0.01–6.37 ± 0.06 mg/100 g, respectively. Among all the derivative amino acids, the amount of carnosine amount was found to be high in chicken meat, whereas α-amino-n-butyric acid was found in low quantities.

Table 2 depicts the free amino acid amounts of essential, non-essential and derivative amino acids in chicken meat samples for different treatments and storage intervals. In the chicken meat control samples, the free amino acid amounts of essential, non-essential and derivative amino acids ranged from 112.61 ± 0.37 to 134.89 ± 0.55, 152.01 ± 0.23 to 168.89 ± 0.37 and 91.9 ± 0.31 to 107.68 ± 41 mg/100 g, respectively, for different storage intervals (0, 7 and 14 days). Following the addition of kale leaf powder to chicken meat, for 1% KLP, the free amino acid amount of essential, non-essential and derivative amino acids ranged from 117.87 ± 0.4, 157.84 ± 0.87 and 102.43 ± 0.4 to 134.07 ± 0.53, 168.89 ± 0.37 and 107.08 ± 0.47 mg/100 g, respectively, whereas for 2% KLP, the free amino acid quantities of essential, non-essential and derivative amino acids ranged from 118.92 ± 043, 159.45 ± 0.31 and 102.30 ± 0.39 to 134.89 ± 0.55, 167.22 ± 1.3 and 107.68 ± 41 mg/100 g, respectively. When the samples of chicken meat were irradiated with 3 kGy doses, the free amino acid quantities of essential, non-essential and derivative amino acids ranged from 112.61 ± 0.37, 152.01 ± 0.23 and 91.9 ± 0.31 to 128.84 ± 0.44, 155.02 ± 0.29 and 100.18 ± 0.49 mg/100 g, respectively. During the different combinations of irradiation and kale leaf powder, a range of free amino acid quantities of essential, non-essential and derivative amino acids was observed. For 1% KLP + 3 kGy, the free amino acid quantities of essential, non-essential and derivative amino acids ranged from 118.6 ± 0.26, 152.75 ± 0.33 and 93.33 ± 0.41 to 131.63 ± 0.47, 157.41 ± 0.49 and 100.59 ± 0.32 mg/100 g, respectively, while the free amino acid quantities of essential, non-essential and derivative amino acids ranged from 119.7 ± 0.8, 153.3 ± 0.35 and 93.67 ± 0.32 to 132.18 ± 0.43, 158.33 ± 0.32 and 100.86 ± 0.31 mg/100 g for 2% KLP + 3 kGy.

### 2.4. Fatty Acids (FA’s) in Irradiated and Kale Leaf-Treated Chicken Meat

The amounts of fatty acids the chicken meat samples were observed to change significantly (*p* ≤ 0.05) during different storage intervals and treatments (irradiation and kale leaf powder). During different storage (0, 7 and 14 days) and treatments, the average amount of saturated fatty acids (SFA), monounsaturated fatty acids (MUFA) and polyunsaturated fatty acids (PUFA) ranged from 41.18 ± 0.07, 35.57 ± 0.1 and 19.98 ± 0.17 to 47.5 ± 1.82, 41.88 ± 0.1 and 31.28 ± 0.23, respectively.

The mean quantity of SFA (fatty acids), C14:0, C16:0, C18:0 and C22:0 ranged from 0.59 ± 0.02–0.76 ± 0.01 to 24.79 ± 0.05–27.98 ± 0.05, 7.03 ± 0.05–10.16 ± 0.04 and 6.05 ± 0.04–9.17 ± 0.05, respectively, following the irradiated and non-irradiated treatments. In this case, the most prevalent free fatty acid was C16:0, whereas the least prevalent free fatty acid was C14:0. The mean quantities of MUFA (fatty acids), C16:1, C18:1n9c and C18:1n9t ranged from 4.25 ± 0.02–5.79 ± 0.01 to 32.07 ± 0.04–35.91 ± 0.05 and 0.21 ± 0.01–0.33 ± 0.01, respectively, following irradiated and non-irradiated treatments. In this case, the most prevalent free fatty acid was C18:1n9c, whereas the least prevalent free fatty acid was C18:1n9t. The mean quantities of PUFA (fatty acids), C18:2n6c, C18:2n6t, C18:3n3, C20:3n6 and C20:4n6 ranged from 12.75 ± 0.04–15.75 ± 0.05 to 11.54 ± 0.03–12.45 ± 0.04, 0.25 ± 0.01–0.64 ± 0.06, 0.40 ± 0.03–0.55 ± 0.02 and 1.41 ± 0.02–1.96 ± 0.05, respectively, following irradiated and non-irradiated treatments. In this case, the most prevalent free fatty acid was C18:2n6c, whereas the least prevalent free fatty acid was C20:3n6.

The quantities of SFA, MUFA and PUFA in chicken meat samples for different treatments and storage intervals are shown in Table 3. SFA, MUFA and PUFA levels in chicken meat control samples ranged from 41.18 ± 0.07, 35.57 ± 0.1 and 19.98 ± 0.17 to 47.5 ± 1.82, 41.88 ± 0.1, and 31.28 ± 0.23 for different storage times (0, 7, and 14 days). Following the addition of kale leaf powder to chicken meat, for 1% KLP, the quantities of saturated fatty acids (SFA), monounsaturated fatty acids (MUFA) and polyunsaturated fatty acids (PUFA) ranged from 42.91 ± 0.13, 39.56 ± 0.08 and 29.78 ± 0.14 to 44.95 ± 0.09, 40.23 ± 0.07 and 30.41 ± 0.19. For 2% KLP, the quantities of SFA, MUFA and PUFA ranged from 42.27 ± 0.15, 40.09 ± 0.08 and 30.47 ± 0.16 to 43.77 ± 0.13, 41.88 ± 0.1 and 31.28 ± 0.23, respectively. When the sample of chicken meat was treated with a radiation dose of 3 kGy, the quantities of SFA, MUFA and PUFA ranged from 43.72 ± 0.38, 35.57 ± 0.1 and 26.64 ± 0.18 to 47.11 ± 0.84, 36.6 ± 0.07 and 27.36 ± 0.19, respectively. During the different combinations of irradiation and kale leaf powder, the quantities of SFA, MUFA and PUFA were observed to change. For 1% KLP + 3 kGy, the quantities of SFA, MUFA and PUFA ranged from 41.3 ± 0.09, 39.11 ± 0.07 and 28.46 ± 0.17 to 42.96 ± 0.1, 39.52 ± 0.11 and 29.03 ± 0.15, respectively, while the quantities of SFA, MUFA and PUFA ranged from 41.18 ± 0.07, 39.54 ± 0.07 and 19.98 ± 0.08 to 41.52 ± 0.17, 39.88 ± 0.07 and 29.17 ± 0.2, respectively, for 2% KLP + 3 kGy.

## 3. Discussions

We noted that the pH value of chicken meat decreases with the increase in kale leaf powder, whereas an increase in pH was observed following an irradiation dose of 3 kGy. The chicken meat was treated with gamma irradiation in the presence of Moringa Leaf Powder (MLP) or without MLP. The pH value was reported to increase with the passage of time and irradiation dose, whereas the minimum PH value was recorded when the meat was treated with MLP [18]. Arshad et al. [19] proved that the pH value increases with an increased irradiation dose. This study was conducted on frozen duck meat and different E-beam doses (0, 3 and 7 kGy) were applied. Similar findings to our results were reported for a study in which smoked duck meat was irradiated with different doses at different storage periods. The pH value was observed to increase with increased E-beam treatment and with increased storage intervals [20]. Horbańczuk and Wierzbicka [21] reported that ostrich meat has a high pH value compared to other meat, which is in line with our findings. A similar pH was recorded in another study in which ostrich meat was packaged different materials [22]. Furthermore, the reported pH of ostrich steaks with different packaging under refrigeration is in agreement with our results [23]. The pH value was found to be higher in ostrich meat compared to beef, game and pork [24]. In a study by Akram et al. [25], it was reported that the pH value of ostrich meat was found to be high compared to goat meat.

Our results showed that the quantity of HAAs increased following the addition of kale leaf powder, while the amount of amino acids reduced following the irradiation dose. Our results confirmed that with the addition of kale leaf powder, the quantities of both groups of amino acids increase, and decline in the amount of amino acids was recorded when the meat was subjected to irradiation. Sikora and Bodziarczyk [26] conducted research on kale and proved that kale is a leafy vegetable and a good source of antioxidants and polyphenols. The enhancement in the level of amino acids in meat and meat products is due to the addition of bioactive compound-containing kale leaves. The study is similar to our work in which a plant extract was added to chicken meat. With the addition of this plant extract, the quantity of essential and non-essential amino acids in the meat was to be observed high. Our results are similar to those reported by Erkan and Özden, [27] who observed that the amount of amino acids in sea bream declined when it was subjected to irradiation. Al-Kahtani et al. [28] irradiated mackerel fish at a dose of 10 kGy. Both essential and non-essential amino acids are the basic building blocks of the human body. They play essential roles in protein formation and also contribute to a host of other intracellular metabolic pathways (from nucleotide synthesis, ATP generation, and redox balance to organismal function and cellular processes). These amino acids aid in the development of immune cells by allowing the pathways to acquire energy and biomass and to reprogram their metabolism [29].

Our results showed that the quantity of free amino acids in chicken meat increased with the addition of kale leaf powder, while the quantities were reduced at different storage intervals. A previous study predicted that fresh and processed kale leaves are good sources of amino acids. Kale leaves contain both essential and non-essential amino acids in vital quantities [30]. Satheesh and Workneh Fanta [31] reported that kale is a rich source of essential and non-essential amino acids. A study was conducted on different types of meat (chicken, turkey, beef, lamb and pork) at various storage intervals. One muscle from a chicken leg, four muscles from turkey, seven muscles from lamb, four muscles from beef and five muscles from pork were collected from a retail outlet. White meat samples (chicken and turkey) contained a high value of total free amino acids, as compared to red meat (lamb, beef and pork), which was similar to our results. On the other hand, during the storage of both red and white meat, the quantities of free amino acids changed, which is also similar to our results [32]. Gómez-Limia et al. [33] processed European eels using different processing techniques and stored them at different intervals. The amounts of essential and non-essential free amino acids changed following 2 months and 12 months of storage. Jiang and Lee [34] predicted that the amount of free amino acids in fish muscle is unstable when frozen in storage.

Our results demonstrated that the amount of fatty acids in meat decreased with increased storage time and irradiation treatment. With the addition of kale leaf powder, the amount of MUFAs and PUFAs was found to be high, while decreasing amounts of SFA were observed with the addition of kale leaf powder. Our results are in agreement with those reported by Giampietro-Ganeco et al. [35], who analyzed the fatty acid contents of raw chicken breasts, drumsticks and thighs. Chicken breast contains a significant amount of fat and cholesterol. Thigh and breast meat contain high levels of omega-3 and omega-6, whereas all chicken meat contains a high amount of PUFA. Recent research analyzed the fat content of ostrich meat in which the muscle of an ostrich was stored for 0, 4, 8, 12, 16 days and packaged in vacuum packaging (VP) and modified atmosphere packaging (MAP). The amount of PUFA was found to vary significantly across storage intervals [36]. Kim et al. [37] reported that during irradiation treatment (4 kGy), the amount of unsaturated fatty acids was found to decrease. This result is in agreement with our findings. Another previous study documented that the irradiation process reduced the polyunsaturated fatty acid content in fresh bonito [38]. Our results are related to the results of a previous study that was conducted using sea bream. When the sample was treated with different irradiation doses, the PUFA quantity was observed to decrease [27]. Kale is composed of different fatty acids in its leaves and seeds. Some fatty acids, such as 16:0, 18:2n-6 and 18:3n-3, are present in higher amounts in kale leaves as compared to seeds. Another fatty acid named linoleic acid is the second most abundantly available fatty acid in kale leaves [39].

## 4. Materials and Methods

### 4.1. Procurement of Raw Material

**Figure 2:**
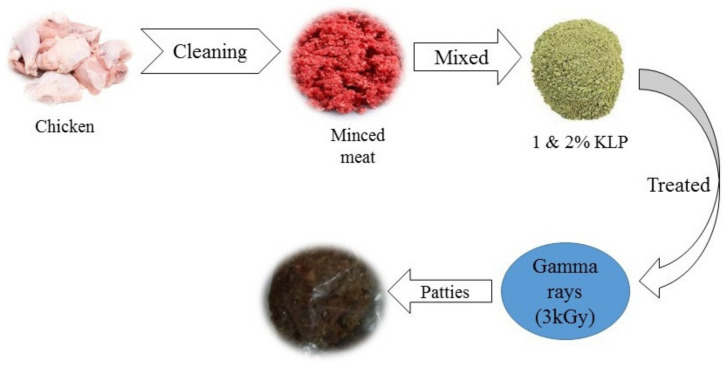
Kale leaf powder- and gamma irradiation-treated chicken patties.

The current study was carried out at the Department of Food Science, Government College University, Faisalabad and at the Nuclear Institute for Agriculture and Biology (NIAB), Faisalabad, Pakistan. The raw chicken meat was obtained from SB Mart Faisalabad, Pakistan. Impermeable plastic films were used to package the chicken meat. In this research, all chemicals and reagents were purchased from Sigma Aldrich (Tokyo, Japan). The chicken patty preparation procedure is shown in Figure 2.

### 4.2. Kale Leaf Powder Preparation

Kale leaves were purchased from METRO Cash and Carry, Faisalabad, Punjab, Pakistan. The raw leaves were washed using deionized water in order to remove undesirable particles and the fresh edible leaves were separated from the inedible leaves. The leaves were then shadow-dried in the laboratory. After this, leaf powder was prepared by grinding the dry leaves and the powder stored in air-tight containers for further analysis. 

### 4.3. Irradiation Treatment

Gamma irradiation treatment was applied to chicken samples at the Nuclear Institute for Agriculture and Biology, Faisalabad, Pakistan under the approval of the Pakistan Atomic Energy Commission. In addition, 3 kGy gamma rays were used with and without KLP.

### 4.4. Storage Intervals

The effects of gamma irradiation and kale leaf powder on chicken meat samples were measured at different intervals (0, 7 and 14 d). The chicken meat samples were aerobically packaged and placed in the refrigerator (4 °C) for different storage durations (7 and 14 d) [40].

### 4.5. pH

A digital pH meter (Model 520A, Orion Research inc., Boston, MA, USA) was used to determine the pH of the chicken meat samples. Chicken meat samples were homogenized with distilled water. At 25 °C, 4.01, 7.00 and 10.01, standard pH buffers were used to calibrate the pH. Three replicates of each sample from chicken meat were used to measure the pH.

### 4.6. Measurement of Amino Acids

#### Hydrolyzed Amino Acids

The amount of hydrolyzed amino acids was measured using the method described by AOAC [41]. For the extraction of hydrolyzed amino acids (HAAs), different types of chicken meat were used. First, a 1 g sample from chicken meat was taken. Each 1 g was hydrolyzed with 15 mL of 6 N HCl for 1 day at 110 °C. At 55 °C, a rotary vacuum evaporator was used to adjust the filtrate concentration after removing the solvent. The remaining materials were diluted using 10 mL of 0.2 N sodium citrate buffer and then a 0.45 μm syringe filter was used for filtration purposes. Hydrolyzed amino acids were expressed in mg/100 g.

### 4.7. Free Amino Acids

The amount of free amino acids in the chicken meat sample was determined according to the method proposed by Simon-Sarkadi and Holzapfel [42], with some modifications. For this purpose, 3 g of meat was taken from the chicken meat. After this, the 3 g meat sample was centrifuged at 7000× *g* for 10 min; then, 15 mL of deionized distilled water was mixed with the meat to ensure it was homogenized. Trichloroacetic acid (12%) from Sigma-Aldrich (Tokyo, Japan) was added to the supernatant and stored for over one hour. After this, it was homogenized using a centrifuge for 20 min. Diethyl ether was used to remove the supernatant with a rotary vacuum evaporator at 40 °C, and at the same time, the lipid phase and trichloroacetic acid were extracted. A 0.2 N sodium citrate buffer (pH 2.2) was used to adjust the 10 mL volume of the solution; then, a 0.45 μm syringe filter (Sartorius, Göttingen, Germany) was used for filtration. The amount of free amino acids was measured using an amino acid analyzer and expressed in mg/100 g.

### 4.8. Determination of Fatty Acid Profile

Fatty acid composition of the chicken meat samples was measured according to the Korean Food Code [43]. A two-gram meat sample was taken from the chicken meat. A solution was made that contained the 2 g sample, 2 mL of ethanol, 10 mL of 8.3 M HCl (anhydrolyzed), 100 mg of pyrogallol and an internal standard solution (triundecanoin 5 mg/mL in chloroform). These components were all mixed together for 40 min at 80 °C. To analyze the amount of total lipids, diethyl ether was used, and nitrogen gas was used to convert the solvent into an evaporated form. Then, the fatty acids in the sample were methylated. The sample was mixed with 4 mL of sodium chloride solution. After this, the solution was kept in a vortex mixer to increase the clarity of the separation phase. Then, 1 mL of hexane, 1.0 g of anhydrous sodium sulfate and 5.0 mL of deionized distilled water were added for the purpose of separation of the solution into aqueous and organic layers. Before using gas chromatography to measure the amount of fatty acids, anhydrous sodium sulfate was added to dehydrate the supernatant. The GC instrument was equipped with a capillary GC column (SP-2560, 100 m × 0.25 mm internal dia) (Superclo Inc., 24056, Bellefonte, PA, USA) and a flame ionization detector (7890; Agilent Technologies, Palo Alto, CA, USA). The conditions for operating the instrument were kept as N_2_ gas (carrier) and a 1 μL injection volume at a 1.0 mL/min flow rate. The injector and detector temperatures were 260 °C and 280 °C, respectively. The initial temperature of the oven was 100 °C for 5 min, while the final temperature of the oven was 230 °C. The temperature of the oven was maintained at 200 °C for 25 min. The temperature of the oven was increased up to 240 °C and kept constant for short time (5 min). Fatty acids in the meat samples were measured by comparing the standard fatty acid methyl ester mixture and chromatogram peak retention times. The quantities of fatty acids, including trans-fatty acids, saturated fatty acids, monounsaturated fatty acids and polyunsaturated fatty acids, were measured for both types of meat.

### 4.9. Statistical Analysis

The results were collected from various parameters that were statistically analyzed by using the statistical package Statistic 8.1 (ANOVA) and CRD to measure the significance level (alpha 5%) [44]. The mean value of different chicken meat samples was compared by using the least significant difference (LSD). Three replicates were used for all parameters.

## 5. Conclusions

It was concluded that the pH, amino acid and fatty acid profiles changed following different treatments (gamma irradiation dose (3 kGy) and kale leaf powder) at different storage intervals. The pH value was increased by the addition of kale leaf powder, whereas the pH value was decreased following the gamma irradiation dose of 3 kGy and the passage of time. The essential and non-essential amino acids found in the chicken meat samples decreased following the irradiation dose of 3 kGy, but these values increased with the addition of kale leaf powder. Moreover, the different storage intervals and irradiation treatment reduced the quantity of fatty acids, such as saturated, monounsaturated and polyunsaturated fatty acids, in the samples, but the quantity of saturated fatty acids further decreased with the addition of kale leaf powder. The chicken meat samples that were treated with 2% KLP showed a slight improvement in the amount of monounsaturated and polyunsaturated fatty acids in the meat.

## Figures and Tables

**Table 1:** Hydrolyzed amino acid (mg/100 g) composition of kale leaf powder- and gamma irradiation-treated chicken meat.

Hydrolyzed Amino Acids	Treatments (Kale Leaf Powder and Gamma Irradiation Dose)					
	Control	1% KLP	2% KLP	3 kGy	1% KLP + 3 kGy	2% KLP + 3 kGy
**Essential**						
Arginine	1069 ± 28	1079 ± 40	1089 ± 43	985 ± 42	993 ± 49	997 ± 53
Histidine	454 ± 18	460 ± 20	466 ± 34	410 ± 15	416 ± 20	422 ± 24
Isoleucine	566 ± 25	572 ± 35	578 ± 40	499 ± 23	507 ± 27	510 ± 26
Leucine	1249 ± 25	1254 ± 45	1259 ± 51	1185 ± 20	1197 ± 23	1205 ± 26
Lysine	1366 ± 20	1372 ± 29	1380 ± 35	1270 ± 19	1274 ± 22	1278 ± 26
Methionine	404 ± 15	409 ± 17	414 ± 24	372 ± 21	378 ±27	382 ± 25
Phenylalanine	671 ± 25	679 ± 20	687 ± 18	623 ± 30	631 ± 29	640 ± 20
Threonine	702 ± 35	708 ± 30	712 ± 28	623 ± 40	634 ± 50	645 ± 48
Valine	578 ± 19	583 ± 23	590 ± 32	517 ± 25	535 ± 24	544 ± 20
Subtotal	7059 ± 210 ^b^	7116 ± 259 ^a,b^	7175 ± 305 ^a^	6487 ± 235 ^e^	6565 ± 271 ^d^	6623 ± 268 ^c^
**Non-Essential**						
Aspartic acid	1559 ± 32	1576 ± 24	1583 ± 20	1499 ± 30	1511 ± 21	1524 ± 26
Alanine	998 ± 20	1006 ± 32	1019 ± 40	982 ± 18	986 ± 34	989 ± 36
Cysteine	61 ± 8	64 ± 6	67 ± 10	42 ± 14	44 ± 15	46 ± 14
Glutamic acid	2376 ± 25	2401 ± 34	2413 ± 30	2343 ± 26	2358 ± 20	2366 ± 18
Glycine	759 ± 24	765 ± 20	778 ± 25	709 ± 18	715 ± 20	725 ± 24
Proline	611 ± 15	620 ± 21	632 ± 35	556 ± 14	598 ± 18	601 ± 20
Serine	628 ± 19	638 ± 24	649 ± 30	574 ± 20	581 ± 26	588 ± 30
Tyrosine	598 ± 14	606 ± 20	610 ± 28	538 ± 32	546 ± 28	554 ± 30
Subtotal	7590 ± 157 ^b^	7676 ± 181 ^a,b^	7721 ± 218 ^a^	7216 ± 172 ^d^	7339 ± 182 ^c^	7393 ±198 ^c^

KLP: Kale leaf powder. The values are mean ± SD of three independent determinations; means with different letters (^a, b, c, d, e^) in a column differed significantly.

**Table 2:** Free amino acid composition (mg/100 g) of chicken meat treated with kale leaf powder and gamma irradiation at different storage intervals.

Treatments (Kale Leaf Powder and Gamma Irradiation Dose)							
Free Amino Acids	Storage Days	Control	1% KLP	2% KLP	3 kGy	1% KLP + 3 kGy	2% KLP + 3 kGy
**Essential**							
Arginine	0	85.73 ± 0.16	86.35 ± 0.15	86.81 ± 0.15	84.59 ± 0.12	86.20 ± 0.16	86.46 ± 0.16
	7	73.18 ± 0.09	74.08 ± 0.09	74.69 ± 0.09	73.13 ± 0.05	73.99 ± 0.05	74.04 ± 0.06
	14	70.51 ± 0.08	71.25 ± 0.06	71.89 ± 0.06	65.69 ± 0.08	71.12 ± 0.05	71.15 ± 0.05
Histidine	0	1.59 ± 0.04	1.62 ± 0.04	1.65 ± 0.05	1.67 0.05	1.70 0.03	1.73 ± 0.03
	7	1.54 ± 0.05	1.60 ± 0.05	1.63 ± 0.03	1.54 ± 0.05	1.53 ± 0.03	1.57 ± 0.09
	14	1.68 ± 0.08	1.61 ± 0.04	1.64 ± 0.06	1.53 ± 0.07	1.42 ± 0.02	1.45 ± 0.03
Isoleucine	0	4.39 ± 0.05	4.46 ± 0.04	4.53 ± 0.05	4.10 ± 0.03	4.39 ± 0.02	4.42 ± 0.02
	7	5.14 ± 0.06	5.15 ± 0.06	5.19 ± 0.04	4.49 ± 0.08	4.65 ± 0.05	4.70 ± 0.04
	14	5.45 ± 0.08	5.48 ± 0.06	5.53 ± 0.05	5.59 ± 0.05	5.50 ± 0.04	5.53 ± 0.05
Leucine	0	9.88 ± 0.07	9.92 ± 0.09	9.95 ± 0.09	9.75 ± 0.05	9.78 ± 0.05	9.81 ± 0.04
	7	11.68 ± 0.08	11.38 ± 0.06	11.44 ± 0.05	10.57 ± 0.02	10.67 ± 0.03	10.73 ± 0.05
	14	11.24 ± 0.06	11.35 ± 0.04	11.42 ± 0.06	11.12 ± 0.03	11.08 ± 0.01	11.13 ± 0.03
Lysine	0	2.69 ± 0.02	2.65 ± 0.02	2.75 ± 0.03	1.91 ± 0.01	1.95 ± 0.01	1.98 ± 0.01
	7	0.99 ± 0.01	1.06 ± 0.02	1.12 ± 0.03	1.37 ± 0.03	1.39 ± 0.03	1.44 ± 0.04
	14	0.45 ± 0.01	0.48 ± 0.01	0.50 ± 0.01	1.18 ± 0.02	1.17 ± 0.02	1.20 ± 0.04
Methionine	0	3.14 ± 0.02	3.19 ± 0.02	3.21 ± 0.02	3.37 ± 0.04	3.41 ± 0.04	3.46 ± 0.05
	7	3.27 ± 0.05	3.42 ± 0.04	3.51 ± 0.03	3.16 ± 0.02	3.50 ± 0.05	3.59 ± 0.05
	14	3.32 ± 0.04	3.73 ± 0.06	3.82 ± 0.04	3.17 ± 0.03	3.24 ± 0.02	3.32 ± 0.02
Phenylalanine	0	6.03 ± 0.06	6.05 ± 0.06	6.08 ± 0.05	5.17 ± 0.04	5.47 ± 0.06	5.77 ± 0.03
	7	5.99 ± 0.05	5.81 ± 0.03	5.84 ± 0.04	5.29 ± 0.06	5.30 ± 0.02	5.33 ± 0.05
	14	6.06 ± 0.04	6.01 ± 0.05	6.06 ± 0.06	5.78 ± 0.05	5.89 ± 0.01	5.99 ± 0.03
Threonine	0	9.95 ± 0.07	9.98 ± 0.06	10.00 ± 0.05	9.29 ± 0.05	9.45 ± 0.04	9.47 ± 0.04
	7	8.69 ± 0.05	8.71 ± 0.04	8.75 ± 0.05	9.05 ± 0.05	9.25 ± 0.05	9.29 ± 0.02
	14	8.29 ± 0.04	8.33 ± 0.03	8.39 ± 0.04	8.19 ± 0.02	9.23 ± 0.05	9.25 ± 0.05
Valine	0	9.79 ± 0.07	9.85 ± 0.05	9.91 ± 0.06	8.99 ± 0.05	9.28 ± 0.06	9.08 ± 0.05
	7	10.72 ± 0.05	10.85 ± 0.08	10.98 ± 0.06	10.58 ± 0.06	10.88 ± 0.03	10.99 ± 0.04
	14	9.45 ± 0.06	9.63 ± 0.05	9.67 ± 0.05	10.36 ± 0.02	10.56 ±0.04	10.68 ± 0.05
Subtotal	0	133.19 ± 0.56 ^A,b^	134.07 ± 0.53 ^A,a^	134.89 ± 0.55 ^A,a^	128.84 ± 0.44 ^A,d^	131.63 ± 0.47 ^A,c^	132.18 ± 0.43 ^A,c^
	7	121.2 ± 0.51 ^B,c^	122.06 ± 0.47 ^B,b^	123.15 ± 0.42 ^B,a^	119.18 ± 0.42 ^B,d^	121.16 ± 0.34 ^B,c^	121.68 ± 0.44 ^B,c^
	14	116.45 ± 0.49 ^C,c^	117.87 ± 0.4 ^C,b^	118.92 ± 043 ^C,a^	112.61 ± 0.37 ^C,d^	118.6 ± 0.26 ^C,a^	119.7 ± 0.80 ^C,a^
**Non-Essential**							
Aspartic acid	0	0.62 ± 0.02	0.65 ± 0.02	0.68 ± 0.02	0.46 ± 0.01	0.49 ± 0.01	0.52 ± 0.02
	7	0.20 ± 0.01	0.21 ± 0.01	0.22 0.02	0.30 ± 0.03	0.34 ± 0.02	0.36 ± 0.02
	14	0.32 ± 0.02	0.33 ± 0.02	0.34 ± 0.01	0.16 ± 0.01	0.23 ± 0.02	0.24 ±0.01
Alanine	0	39.85 ± 0.10	39.96 ± 0.12	40.01 ± 1.02	36.12 ± 0.09	36.99 ± 0.08	37.08 ± 0.08
	7	38.83 ±0.10	38.94 ± 0.11	39.01 ± 0.09	38.07 ± 0.08	38.36 ± 0.07	38.43 ± 0.09
	14	38.04 ± 0.10	38.10 ± 0.08	39.13 ± 0.09	34.99 ± 0.06	35.00 ± 0.07	35.03 ± 0.08
Cysteine	0	0.59 ± 0.02	0.63 ± 0.02	0.65 ± 0.03	0.50 ± 0.02	0.53 ± 0.02	0.61 ± 0.01
	7	0.48 ± 0.01	0.49 ± 0.01	0.51 ± 0.02	0.61 ± 0.01	0.62 ± 0.02	0.64 ± 0.01
	14	0.34 ± 0.02	0.37 ± 0.03	0.39 ± 0.02	0.43 ± 0.01	0.46 ± 0.01	0.49 ± 0.03
Glutamic acid	0	70.46 ± 0.12	70.72 ± 0.10	70.84 ± 0.09	68.01 ± 0.06	68.13 ± 0.03	68.22 ± 0.03
	7	68.55 ± 0.09	68.67 ± 0.08	68.71 ± 0.06	67.16 ± 0.05	67.39 ± 0.04	67.44 ± 0.04
	14	68.97 ± 0.09	68.95 ± 0.08	69.11 ± 0.06	67.91 ± 0.07	67.65 ± 0.0.06	67.70 ± 0.08
Glycine	0	14.53 ± 0.05	14.71 ± 0.04	14.87 ± 0.05	13.01 ± 0.03	13.40 ± 0.02	13.63 ±0.04
	7	14.02 ± 0.02	14.10 ± 0.05	14.15 ± 0.04	13.10 ± 0.03	13.67 ± 0.06	13.87 ± 0.04
	14	14.32 ± 0.03	14.58 ± 0.04	14.65 ± 0.03	13.65 ± 0.02	13.99 ± 0.06	14.07 ± 0.05
Proline	0	20.75 ± 0.06	21.04 ± 0.03	21.22 ± 0.05	18.73 ± 0.03	19.01 ± 0.06	19.10 ± 0.07
	7	20.18 ± 0.05	20.24 ± 0.06	20.51 ± 0.06	19.65 ± 0.05	19.87 ± 0.04	19.94 ± 0.05
	14	20.89 ± 0.06	21.01 ± 0.05	21.20 ± 0.04	19.91 ± 0.03	19.99 ± 0.06	20.19 ± 0.03
Serine	0	12.41 ± 0.05	12.50 ± 0.01	12.66 ± 0.02	12.75 ± 0.04	12.54 ± 0.02	12.82 ± 0.01
	7	10.57 ± 0.04	10.76 ± 0.06	10.80 ± 0.05	10.88 ± 0.04	10.90 ± 0.05	10.93 ± 0.04
	14	10.59 ± 0.04	10.53 ± 0.05	10.63 ± 0.03	10.59 ± 0.01	10.78 ± 0.02	10.83 ± 0.05
Tyrosine	0	6.53 ± 0.02	6.68 ± 0.03	6.79 ± 0.02	5.32 ± 0.01	6.32 ± 0.02	6.40 ± 0.03
	7	4.47 ± 0.02	4.73 ± 0.04	4.78 ± 0.03	4.02 ± 0.03	5.36 ± 0.01	5.42 ± 0.03
	14	3.72 ± 0.03	3.97 ± 0.02	4.00 ± 0.03	4.37 ± 0.02	4.65 ± 0.03	4.75 ± 0.02
Subtotal	0	165.74 ± 0.44 ^A,b^	168.89 ± 0.37 ^A,a^	167.22 ± 1.3 ^A,a^	155.02 ± 0.29 ^A,d^	157.41 ± 0.26 ^A,c^	158.33 ± 0.56 ^A,c^
	7	157.3 ± 0.34 ^B,a^	158.14 ± 0.87 ^B,a^	158.69 ± 0.91 ^B,a^	153.79 ± 0.32 ^B,d^	156.51 ± 0.49 ^B,c^	157.03 ± 0.32 ^B,b^
	14	157.19 ± 0.39 ^B,b^	157.84 ± 0.87 ^B,b^	159.45 ± 0.31 ^B,a^	152.01 ± 0.23 ^C,c^	152.75 ± 0.33 ^C,c^	153.3 ± 0.35 ^C,c^
**Derivatives**							
Anserine	0	1.28 ± 0.01	1.30 ± 0.01	1.32 ± 0.02	1.19 ± 0.01	1.28 ± 0.02	1.29 ± 0.02
	7	1.17 ± 0.01	1.20 ± 0.01	1.23 ± 0.02	1.14 ± 0.01	1.18 ± 0.01	1.21 ± 0.02
	14	1.18 ± 0.01	1.20 ± 0.01	1.21 ± 0.02	1.17 ± 0.01	1.19 ± 0.01	1.20 ± 0.02
Carnosine	0	87.35 ± 0.12	87.60 ± 0.10	87.66 ± 0.05	80.97 ± 0.10	81.09 ± 0.06	81.16 ± 0.05
	7	86.98 ± 0.09	87.19 ± 0.08	87.23 ± 0.05	78.29 ± 0.09	78.57 ± 0.05	78.64 ± 0.08
	14	86.89 ± 0.08	87.28 ± 0.10	87.34 ± 0.09	78.22 ± 0.07	78.48 ±0.08	78.55 ± 0.05
Cystathionine	0	1.48 ± 0.05	1.50 ± 0.04	1.51 ± 0.03	1.35 ± 0.05	1.38 ± 0.04	1.41 ±0.05
	7	1.39 ± 0.04	1.36 ± 0.03	1.38 ±0.04	1.29 ± 0.01	1.31 ± 0.02	1.32 ± 0.02
	14	1.21 ± 0.02	1.23 ± 0.02	1.25 ± 0.02	1.15 ± 0.01	1.19 ± 0.01	1.20 ± 0.02
Ethanol amine	0	0.39 ± 0.01	0.49 ± 0.02	0.51 ± 0.01	0.50 ± 0.02	0.54 ± 0.01	0.56 ± 0.02
	7	0.99 ± 0.03	1.04 ± 0.04	1.07 ± 0.04	0.80 ± 0.03	0.85 ± 0.02	0.87 ± 0.01
	14	1.88 ± 0.04	1.88 ± 0.04	1.24 ± 0.03	0.91 ± 0.02	0.91 ± 0.02	0.93 ± 0.01
Hydroxy proline	0	2.98 ± 0.05	3.04 ± 0.06	3.06 ± 0.04	3.21 ± 0.05	3.31 ± 0.01	3.34 ± 0.02
	7	3.12 ± 0.06	3.14 ± 0.03	3.16 ± 0.03	3.12 ± 0.01	3.09 ± 0.06	3.11 ± 0.02
	14	2.99 ± 0.03	3.03 ± 0.04	3.05 ± 0.02	3.01 ± 0.02	3.07 ± 0.04	3.09 ± 0.03
Sarcosine	0	0.40 ± 0.02	0.42 ± 0.02	0.43 ± 0.01	0.32 ± 0.03	0.35 ± 0.02	0.37 ± 0.02
	7	0.36 ± 0.01	0.37 ± 0.02	0.39 ± 0.02	0.25 ± 0.01	0.34 ± 0.02	0.35 ± 0.01
	14	0.33 ± 0.01	0.35 ± 0.01	0.37 ± 0.02	0.23 ± 0.01	0.30 ± 0.02	0.31 ± 0.02
α-Amino adipic acid	0	1.63 ± 0.04	1.72 ± 0.05	1.81 ± 0.05	1.46 ± 0.06	1.60 ± 0.05	1.68 ± 0.02
	7	1.37 ± 0.06	1.40 ± 0.04	1.42 ± 0.05	1.46 ± 0.04	1.39 ± 0.03	1.52 ± 0.02
	14	1.35 ± 0.05	1.36 ± 0.04	1.37 ± 0.05	1.43 ± 0.02	1.38 ± 0.03	1.47 ± 0.01
α-Amino-n-butyric acid	0	0.27 ± 0.01	0.29 ± 0.01	0.31 ± 0.02	0.22 ± 0.01	0.26 ± 0.01	0.27 ± 0.01
	7	0.24 ± 0.02	0.25 ± 0.02	0.26 ± 0.02	0.19 ± 0.01	0.23 ± 0.01	0.25 ± 0.02
	14	0.21 ± 0.01	0.23 ± 0.01	0.25 ± 0.02	0.22 ± 0.01	0.28 ± 0.02	0.29 ± 0.02
β-Alanine	0	2.28 ± 0.05	2.30 ± 0.05	2.31 ± 0.04	2.21 ± 0.03	2.26 ± 0.03	2.28 ± 0.02
	7	2.26 ± 0.04	2.30 ± 0.05	2.32 ± 0.03	2.31 ± 0.02	2.31 ± 0.02	2.39 0.03
	14	0.23 ± 0.02	2.28 ± 0.04	2.29 ± 0.04	2.29 ± 0.04	2.34 ± 0.05	2.35 ± 0.03
γ-Amino-n-butyricacid	0	0.50 ± 0.01	0.56 ± 0.02	0.59 ± 0.02	0.48 ± 0.01	0.54 ± 0.01	0.57 ± 0.02
	7	0.90 ± 0.02	0.90 ± 0.02	0.93 ± 0.03	0.93 ± 0.03	0.92 ± 0.01	1.05 ± 0.04
	14	1.29 ± 0.04	1.31 ± 0.04	1.33 ± 0.01	1.04 ± 0.03	1.03 ± 0.03	1.04 ± 0.02
1-Methylhistidine	0	2.20 ± 0.05	2.23 ± 0.04	2.25 ± 0.04	2.17 ± 0.06	2.22 ± 0.04	2.24 ± 0,03
	7	2.02 ± 0.04	2.04 ± 0.03	2.05 ± 0.02	2.09 ± 0.04	2.10 ± 0.02	2.15 ± 0.01
	14	1.04 ± 0.03	1.09 ± 0.05	1.11 ± 0.04	1.83 ± 0.06	1.85 ± 0.05	1.89 ± 0.04
3-Methylhistidine	0	5.99 ± 0.06	5.63 ± 0.05	5.65 ± 0.04	6.10 ± 0.06	5.66 ± 0.04	5.69 ± 0.03
	7	5.14 ± 0.05	6.21 ± 0.06	6.24 ± 0.06	5.23 ± 0.05	5.34 ± 0.04	6.37 ± 0.06
	14	0.34 ± 0.01	1.19 ± 0.03	1.22 ± 0.03	0.40 ± 0.0.1	1.31 ± 0.05	1.35 ± 0.05
Subtotal	0	106.75 ± 0.48 ^A,b^	107.08 ± 0.47 ^A,a^	107.41 ± 0.37 ^A,a^	100.18 ± 0.49 ^A,c^	100.59 ± 0.32 ^A,c^	100.86 ± 0.31 ^A,c^
	7	105.94 ± 0.46 ^B,b^	107.4 ± 0.43 ^A,a^	107.68 ± 41 ^A,a^	97.1 ± 0.35 ^B,d^	97.63 ± 0.31 ^B,d^	99.23 ± 0.34 ^B,c^
	14	103.74 ± 0.35 ^C,a^	102.43 ± 0.4 ^B,a^	102.30 ± 0.39 ^B,a^	91.9 ± 0.31 ^C,c^	93.33 ± 0.41 ^C,b^	93.67 ± 0.32 ^C,b^

KLP: Kale leaf powder. The values are mean ± SD of three independent determinations; Capital letters (^A, B, C^) indicates that the values differ significantly within columns, whereas a small letters (^a, b, c, d^) indicates that the values differ significantly within rows.

**Table 3:** Fatty acid composition (%) of chicken meat treated with kale leaf powder and gamma irradiation at different storage intervals.

Fatty Acids	Storage Days	Treatments (Kale Leaf Powder and Gamma Irradiation Dose)					
		Control	1% KLP	2% KLP	3 kGy	1% KLP + 3 kGy	2% KLP + 3 kGy
**SFA**							
C14:0	0	0.74 ± 0.02	0.69 ± 0.01	0.64 ± 0.02	0.72 ± 0.01	0.62 ± 0.03	0.60 ± 0.01
	7	0.71 ± 0.02	0.67 ± 0.01	0.63 ± 0.02	0.76 ± 0.01	0.66 ± 0.03	0.64 ± 0.01
	14	0.72 ± 0.02	0.62 ± 0.03	0.59 ± 0.02	0.74 ± 0.02	0.64 ± 0.03	0.62 ± 0.01
C16:0	0	26.98 ± 0.09	27.33 ± 0.07	27.69 ± 0.06	25.02 ± 0.07	27.07 ± 0.06	27.52 ± 0.05
	7	25.32 ± 0.05	27.40 ± 0.06	27.79 ± 0.06	27.98 ± 0.05	27.44 ± 0.03	27.79 ± 0.02
	14	24.99 ± 0.02	25.95 ± 0.04	26.89 ± 0.04	24.79 ± 0.05	27.01 ± 0.03	27.14 ± 0.01
C18:0	0	10.16 ± 0.04	8.81 ± 0.02	8.05 ± 0.05	9.69 ± 0.03	7.99 ± 0.02	7.15 ± 0.01
	7	9.97 ± 0.03	8.89 ± 0.01	8.17 ± 0.02	9.69 ± 0.03	7.10 ± 0.01	7.03 ± 0.05
	14	9.83 ± 0.01	8.65 ± 0.03	7.89 ± 0.05	9.59 ± 0.03	8.15 ± 0.01	7.20 ± 0.03
C22:0	0	9.17 ± 0.05	7.91 ± 0.02	7.05 ± 0.04	8.68 ± 0.03	6.99 ± 0.04	6.25 ± 0.01
	7	8.99 ± 0.04	7.99 ± 0.01	7.18 ± 0.03	8.68 ± 0.03	6.10 ± 0.02	6.05 ± 0.04
	14	8.87 ± 0.02	7.69 ± 0.03	6.90 ± 0.04	8.60 ± 0.03	7.16 ± 0.03	6.22 ± 0.02
**MUFA**							
C16:1	0	5.21 ± 0.04	5.30 ± 0.02	5.79 ± 0.01	4.25 ± 0.02	5.25 ± 0.01	5.50 ± 0.03
	7	5.17 ± 0.01	5.28 ± 0.03	5.77 ± 0.02	4.60 ± 0.01	5.40 ± 0.03	5.65 ± 0.03
	14	5.14 ± 0.02	5.24 ± 0.03	5.74 ± 0.04	4.34 ± 0.03	5.23 ± 0.02	5.48 ± 0.01
C18:1n9c	0	33.49 ± 0.08	34.07 ± 0.05	34.69 ± 0.06	32.07 ± 0.04	33.99 ± 0.03	34.09 ± 0.05
	7	32.41 ± 0.07	34.01 ± 0.03	34.07 ± 0.06	30.99 ± 0.05	33.87 ± 0.05	34.01 ± 0.03
	14	33.70 ± 0.05	34.75 ± 0.02	35.91 ± 0.05	30.72 ± 0.05	33.65 ± 0.04	33.85 ± 0.05
C18:1n9t	0	0.33 ± 0.01	0.31 ± 0.02	0.29 ± 0.01	0.28 ± 0.01	0.27 ± 0.02	0.24 ± 0.01
	7	0.30 ± 0.01	0.27 ± 0.02	0.25 ± 0.02	0.30 ± 0.01	0.25 ± 0.03	0.22 ± 0.01
	14	0.27 ± 0.01	0.24 ± 0.02	0.23 ± 0.01	0.31 ± 0.02	0.23 ± 0.01	0.21 ± 0.01
**PUFA**							
C18:2n6c	0	14.98 ± 0.03	15.22 ± 0.02	15.75 ± 0.05	13.01 ± 0.03	14.45 ± 0.02	15.05 ± 0.04
	7	14.87 ± 0.03	15.10 ± 0.04	15.62 ± 0.05	12.95 ± 0.04	14.31 ± 0.03	14.95 ± 0.06
	14	14.69 ± 0.02	15.03 ± 0.03	15.40 ± 0.05	12.75 ± 0.04	14.28 ± 0.06	14.65 ± 0.07
C18:2n6t	0	12.38 ± 0.03	12.40 ± 0.04	12.45 ± 0.04	11.85 ± 0.04	11.96 ± 0.03	12.01 ± 0.02
	7	12.24 ± 0.02	12.26 ± 0.03	12.28 ± 0.02	11.54 ± 0.03	11.86 ± 0.04	11.88 ± 0.02
	14	12.20 ± 0.02	12.24 ± 0.03	12.27 ± 0.04	11.65 ± 0.05	11.73 ± 0.04	11.75 ± 0.06
C18:3n3	0	0.52 ± 0.03	0.60 ± 0.04	0.64 ± 0.06	0.50 ± 0.04	0.54 ± 0.05	0.58 ± 0.03
	7	0.30 ± 0.03	0.49 ± 0.04	0.53 ± 0.02	0.32 ± 0.04	0.47 ± 0.05	0.51 ± 0.02
	14	0.25 ± 0.01	0.45 ± 0.04	0.50 ± 0.03	0.31 ± 0.05	0.43 ± 0.04	0.47 ± 0.03
C20:3n6	0	0.55 ± 0.02	0.50 ± 0.01	0.48 ± 0.03	0.52 ± 0.02	0.46 ± 0.01	0.42 ± 0.02
	7	0.52 ± 0.01	0.47 ± 0.02	0.44 ± 0.01	0.50 ± 0.01	0.43 ± 0.02	0.41 ± 0.03
	14	0.53 ± 0.01	0.43 ± 0.02	0.41 ± 0.01	0.48 ± 0.02	0.41 ± 0.01	0.40 ± 0.03
C20:4n6	0	1.50 ± 0.06	1.69 ± 0.08	1.96 ± 0.05	1.48 ± 0.06	1.67 ± 0.04	1.92 ± 0.06
	7	1.44 ± 0.04	1.59 ± 0.04	1.82 ± 0.03	1.41 ± 0.02	1.56 ± 0.03	1.85 ± 0.02
	14	1.47 ± 0.03	1.63 ± 0.02	1.89 ± 0.03	1.45 ± 0.02	1.61 ± 0.02	1.90 ± 0.01
Ʃ SFA	0	47.5 ± 1.82 ^A,a^	44.74 ± 0.12 ^A,b^	43.43 ± 0.16 ^A,b^	44.11 ± 0.14 ^B,b^	42.67 ± 0.15 ^A,c^	41.52 ± 0.08 ^A,d^
	7	44.99 ± 0.14 ^B,b^	44.95 ± 0.09 ^A,b^	43.77 ± 0.13 ^A,c^	47.11 ± 0.84 ^A,a^	41.3 ± 0.09 ^B,d^	41.51 ± 0.12 ^A,d^
	14	44.41 ± 0.07 ^b,a^	42.91 ± 0.13 ^B,c^	42.27 ± 0.15 ^B,c^	43.72 ± 0.38 ^B,b^	42.96 ± 0.1 ^A,c^	41.18 ± 0.07 ^A,d^
Ʃ MUFA	0	39.03 ± 0.13 ^A,b^	39.68 ± 0.09 ^B,b^	40.77 ± 0.08 ^B,a^	36.6 ± 0.07 ^A,c^	39.51 ± 0.06 ^A,b^	39.83 ± 0.09 ^A,b^
	7	37.88 ± 0.09 ^B,c^	39.56 ± 0.08 ^B,b^	40.09 ± 0.1 ^B,a^	35.89 ± 0.07 ^B,d^	39.52 ± 0.11 ^A,b^	39.88 ± 0.07 ^A,b^
	14	39.11 ± 0.08 ^A,c^	40.23 ± 0.07 ^A,b^	41.88 ± 0.1 ^A,a^	35.57 ± 0.1 ^B,d^	39.11 ± 0.07 ^B,c^	39.54 ± 0.07 ^B,c^
Ʃ PUFA	0	29.93 ± 0.17 ^A,c^	30.41 ± 0.19 ^A,b^	31.28 ± 0.23 ^A,a^	27.36 ± 0.19 ^A,d^	29.03 ± 0.15 ^A,c^	19.98 ± 0.17 ^B,d^
	7	29.37 ± 0.01 ^B,b^	29.91 ± 0.17 ^B,b^	30.69 ± 0.13 ^B,a^	26.72 ± 0.16 ^B,d^	28.63 ± 0.17 ^B,c^	29.6 ± 0.15 ^A,b^
	14	29.14 ± 0.09 ^C,b^	29.78 ± 0.14 ^B,b^	30.47 ± 0.16 ^B,a^	26.64 ± 0.18 ^B,d^	28.46 ± 0.17 ^B,c^	29.17 ± 0.2 ^A,b^

KLP: Kale leaf powder. SFA: Saturated fatty acids; MUFA: monounsaturated fatty acids; PUFA: polyunsaturated fatty acids. The values are mean ±SD of three independent determinations; Capital letters (^A, B, C^) indicates that the values differ significantly within columns, whereas small letters (^a, b, c, d^) indicates that the values differ significantly within rows.

## Data Availability

Not applicable.

## Sample Availability

Samples of the compounds are available from the authors.
